# Spatial transcriptomic interrogation of the tumour-stroma boundary in a 3D engineered model of ameloblastoma

**DOI:** 10.1016/j.mtbio.2023.100923

**Published:** 2023-12-21

**Authors:** Deniz Bakkalci, Georgina Al-Badri, Wei Yang, Andy Nam, Yan Liang, Syed Ali Khurram, Susan Heavey, Stefano Fedele, Umber Cheema

**Affiliations:** a UCL Centre for 3D Models of Health and Disease, Division of Surgery and Interventional Science, University College London, Charles Bell House, 43-45 Foley Street, W1W 7TS, London, UK; b Department of Mathematics, University College London, 25 Gordon Street, WC1H 0AY, London, UK; c NanoString Technologies, 530 Fairview Ave N, Seattle, WA 98109, USA; d Unit of Oral and Maxillofacial Pathology, School of Clinical Dentistry, University of Sheffield, 19 Claremont Crescent, S10 2TA, Sheffield, UK; e Eastman Dental Institute, University College London, London, UK

**Keywords:** Spatial transcriptomics, 3D models, Tumour-stroma boundary, Tissue engineering

## Abstract

Stromal cells are key components of the tumour microenvironment (TME) and their incorporation into 3D engineered tumour-stroma models is essential for tumour mimicry. By engineering tumouroids with distinct tumour and stromal compartments, it has been possible to identify how gene expression of tumour cells is altered and influenced by the presence of different stromal cells. Ameloblastoma is a benign epithelial tumour of the jawbone. In engineered, multi-compartment tumouroids spatial transcriptomics revealed an upregulation of oncogenes in the ameloblastoma transcriptome where osteoblasts were present in the stromal compartment (bone stroma). Where a gingival fibroblast stroma was engineered, the ameloblastoma tumour transcriptome revealed increased matrix remodelling genes. This study provides evidence to show the stromal-specific effect on tumour behaviour and illustrates the importance of engineering biologically relevant stroma for engineered tumour models. Our novel results show that an engineered fibroblast stroma causes the upregulation of matrix remodelling genes in ameloblastoma which directly correlates to measured invasion in the model. In contrast the presence of a bone stroma increases the expression of oncogenes by ameloblastoma cells.

## Graphical abstract

**Figure:**
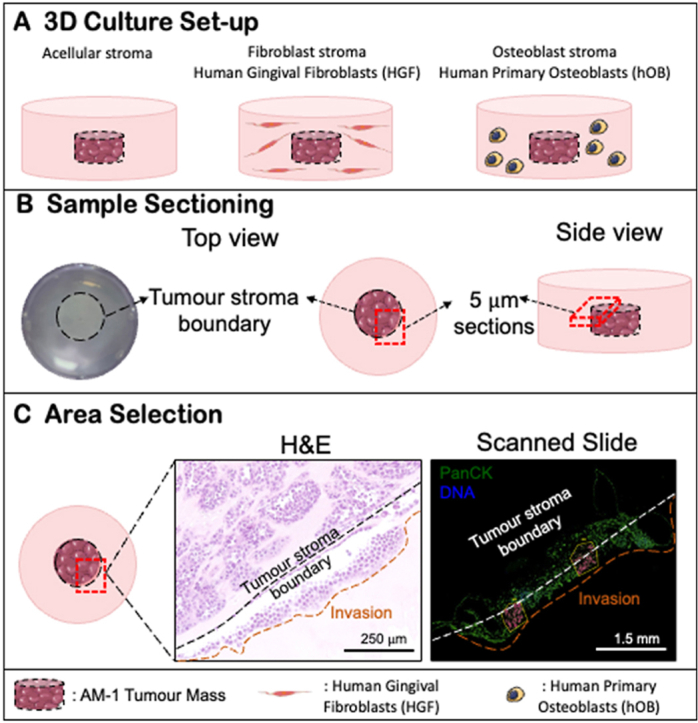

## 1 Introduction

The tumour microenvironment (TME) provides essential cues to direct and control tumour progression and provide the basis of key hallmarks of cancer [1]. The complexity of the TME derives from both cellular and noncellular components. Cellular components are namely stromal cells including fibroblasts, immune cells, bone and endothelial cells, and non-cellular components include the extracellular matrix components, oxygenation of the tissue and cytokines [2]. Biomimetic extracellular matrix (ECM) composition and stromal cells are required to recreate the TME in engineered tissue models [3]. Several different techniques have been studied to mimic cell-cell interactions in the TME both *in vitro* and *in vivo*. Two-dimensional (2D) monolayer co-cultures are widely used as *in vitro* TME models, however they lack many physiological properties, including the tissue architecture. The use of 3D models has made it possible to recapitulate the physiological conditions found in the TME. Furthermore, multicellular 3D models have overcome some of the limitations of 2D model counterparts [4,5].

One of the challenges of multicellular 3D models is the uncertainty around deciphering cell-specific signalling, in other words how cells synchronise their signalling and network with other cells [5]. Thus, novel strategies to unravel this complex communication between tumour and stroma include rapidly evolving technology in high-plex profiling for the development of molecular spatial profiling. The spatial profiling allows cell-type specific characterisation of heterogenous cell populations in the TME. GeoMx Digital Spatial Profiler (DSP) is a platform to spatially resolve biology of the tissue of interest by using digital quantitation of target analytes [6,7]. This system utilises barcoded DNA oligos attached to *in situ* hybridisation probes for RNA. The attachment is done by a detection reagent, an ultraviolet (UV)-photocleavable linker [8]. The tissue is covered with the detection reagent and the customizable fluorescent morphology markers and then visualised. The regions of interest (ROIs) are used to image the sample following UV light exposure-induced release of the barcoded oligos. These oligos can then be collected by instrument quantitation using nCounter or next generation sequencing (NGS) [8]. The ability to select regions of interest means that the interaction of tumour and stroma can be visually dissected on a section and interrogated using this technology.

The GeoMx DSP works well with small sample size and the process itself is non-destructive, therefore the same section can be profiled multiple times [7]. The ROI selection is adjusted based on research of interest. It has been used to study various neoplasms including lung, prostate, breast, and liver cancers and allows for a high level of characterisation of heterogeneous tissue. To our knowledge, this technique has not been successfully utilised in any bioengineered in *vitro* samples, including odontogenic tumours, but the use of a spatial transcriptomics platform will give insight and demystify the crosstalk between tumour and stroma cells. This study utilises GeoMx DSP to spatially resolve tumour-stroma interaction in *vitro* 3D tumouroids of ameloblastoma and their native stromal cells.

Ameloblastoma (AM) is a benign odontogenic tumour of the jawbone, which is rare but locally aggressive [9]. Ameloblastoma tumour cells interact with the bone and gingival fibroblast stroma, leading to resorption of the surrounding maxillofacial jawbones [10]. These interactions regulate the development and progression of the disease, and it is essential to understand the precise mechanisms. Studies on 3D ameloblastoma tumouroids have provided novel findings related to disease mechanism [11].

The use of plastic compression to generate tissue dense collagen scaffolds is a key innovation in developing biomimetic tissue models [12,13]. The plastic compression technique is applied to cell-seeded collagen hydrogels to expel excess fluid, without any loss in cell viability. Plastic compression results in a significant increase in collagen density and an increased stiffness, or youngs modulus, so that collagen scaffolds more closely mimic native human tissue values [14,15]. The tumouroid model generated using plastic compression also allow for compartmentalisation of the tumour and stroma in tumouroids [16].

Through the careful bioengineering of a connected tumour and stroma compartment, where biophysical features of tissue are re-capitulated, it is possible to study the boundary between these components to profile key pathways in tumour cells which are altered in the presence of and through the interaction with specific stromal cells. The pathways analysed in this study were chosen based on our pre-existing gene data [16]. Using tumouroid models of ameloblastoma and other cancers, we have already validated invasive marker genes such as matrix metalloproteinases (MMPs) [11,15]. Due to the large volume of data generated, this study carefully analyses previous data generated using tumouroid-stroma models to focus on developing clear research questions. These are namely ECM remodelling, invasion, and immune regulation.

This powerful model is fully appreciated when focused regions of interest and cell specific analysis can be conducted at the tumour-stroma interface. By using GeoMx DSP it is possible to analyse key cell populations at this interface and investigate larger cohorts of different markers.

## 2 Methods

### 2.1 Cell culture

Cell culture conditions were 37 °C, 5 % CO_2_, and 21 % O_2_. The immortalised plexiform ameloblastoma cell line, AM-1 was provided by Professor Harada [17]. Keratinocyte serum free medium 1*X* (KSFM) supplemented with KSFM supplements (bovine pituitary extract (BPE) and epidermal growth factor (EGF), human recombinant) was used to culture the AM-1 cells. Primary gingival fibroblasts, Human, Adult (HGF) (PCS-201-018™) were purchased from ATCC and cultured in Dulbecco's modified Eagle medium (DMEM). Primary human osteoblasts (hOB) from Promocell® (Heidelberg, Germany) were cultured in Promocell® osteoblast growth medium with supplement mix. All media types contained 10 % foetal bovine serum (FBS), 100 units/mL penicillin, and 100 μg/mL streptomycin (Gibco™ through Thermo Fisher Scientific, Loughborough, UK).

### 2.2 3D model fabrication

3D models were engineered using monomeric type I collagen (First Link, Birmingham, UK). and RAFT™ protocol was followed throughout the process. A collagen/cell mix was prepared from 10*X* Minimal Essential Medium (MEM) (Sigma-Aldrich, Dorset, UK), collagen type I, neutralising agent (N.A) and the cells. N.A was composed of 17 % 10 Molar NaOH (Sigma-Aldrich, Dorset, UK) and 83 % 10 M HEPES buffer (Gibco™ through Thermo Fisher Scientific, Loughborough). The collagen/cell mix had final volumes of 80 % collagen, 10*X* MEM, 6 % N.A. and 4 % cells and was kept on ice until it was crosslinked.

**Fig. 1:**
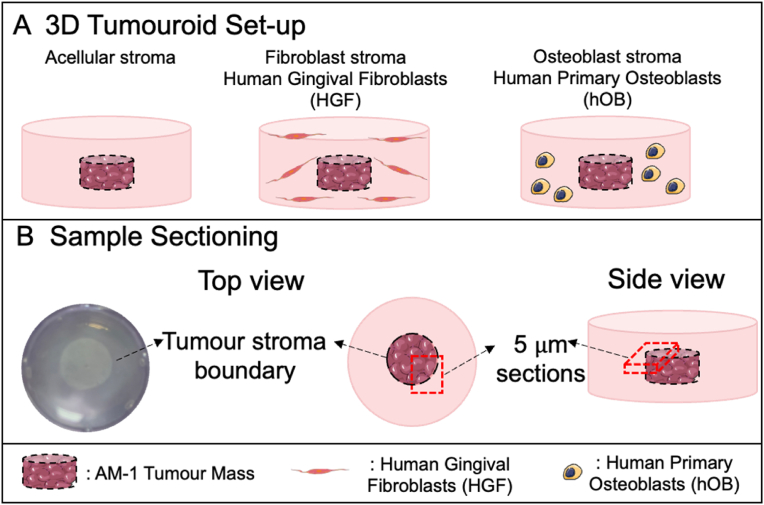
3D model set-up for GeoMx DSP. (A) The 3D tumouroid set-up and (B) sectioning of the 3D tumouroids for GeoMx DSP. Diagrams were created using Smart Servier Medical Art.

The first step in the fabrication of the complex tumouroids was creating the tumour mass of 240 μL of cell/collagen mix with 5 × 10^4^ AM-1 cells. The mix for the tumour mass was then set into 96-well plates (Corning® Costar®, Sigma-Aldrich, Dorset, UK) and incubated in 37 °C for 15 min to allow crosslinking. This step was followed by 15 min of plastic compression using RAFT™ absorbers at room temperature (Lonza, Slough, UK). Then the stromal gel mixes containing either no cells (acellular) or 1 × 10^5^ HGFs or 1 × 10^5^ hOBs were prepared. Cells and collagen were mixed thoroughly to ensure an even distribution of cells throughout the 3D matrix. The first layer, 650 μL of the stromal gel mix was cast on 24-well plate (Corning® Costar ®, Sigma-Aldrich, Dorset, UK). The tumour mass was placed in the middle of the first stromal layer and covered by a second stromal layer of 650 μL of the stromal gel mix (Fig. 1A).

Following crosslinking of the tumouroids for 15 min at 37 °C, 24-well RAFT™ absorbers (Lonza, Slough, UK) were used to plastic compress them for 15 min. The gels were supplied with 2 mL of media, which was changed by 50 % every 48 h. The culture period was 14 days.

### 2.3 Sample preparation for spatial profiler

The protocol from Ref. [8] ‘GeoMxTm RNA Assay: High Multiplex, Digital, Spatial Analysis of RNA in FFPE Tissue’ was followed throughout. The sample preparation section was specific to tissue samples. Therefore, this study has established the sample preparation steps for 3D *in vitro* samples.

3D samples were formalin fixed and processed using a processor (Thermo Fisher Scientific, Loughborough, UK) and embedded. The embedded blocks were sectioned into 5 μm sections and these were trimmed appropriately to mount to the VWR Superfrost Plus Slides (Catalogue number 48311–703). The tumour-stroma boundary was visible by eye, therefore during sectioning the area covering tumour-stroma boundary was targeted (Fig. 1B). From each sample, minimum of 4 sections were collected to maximise the number of cells captured in each slide due to the fact that the cells were not distributed evenly. The invasion from tumour stroma boundary was confirmed by Haematoxylin and eosin (H&E) staining *(*Supplemental Figure 1A*).* The sample was 200 μm and the thickness of the tumour mass was 100 μm. The sections were selected from the tumour mass alignment *(*Supplemental Figure 1B*).* The sections were placed within a defined area (36.2 mm long x 14.6 mm wide) in the middle of the slide.

### 2.4 Selection of region of interest

**Fig. 2:**
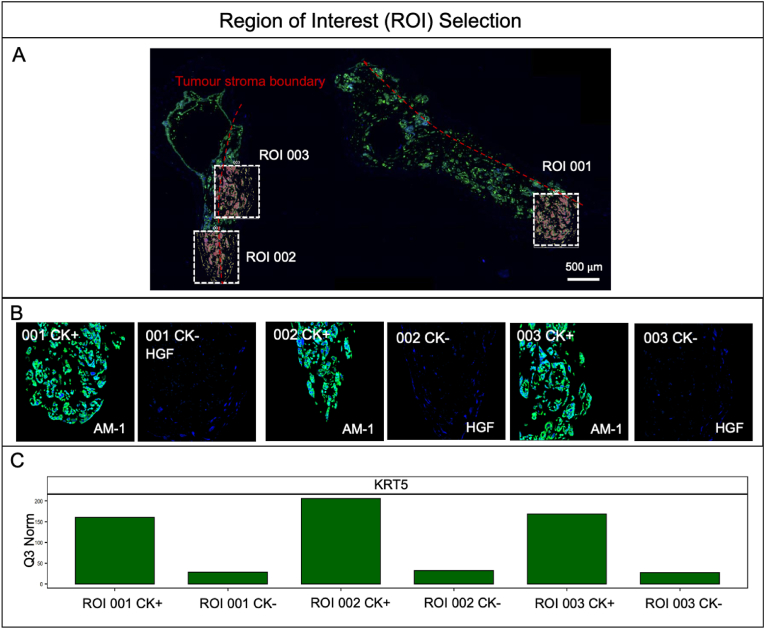
Region of Interest (ROI) selection. (A) Scanned slide of 3D AM-1 tumouroids with HGF stroma and three ROIs, ROI 001, 002, and 003 selected within, scale bar = 500 μm. (B) Cytokeratin-positive (CK+) and CK- sections of ROI 001, 002, and 003. (C) Relative mRNA expression of Keratin 5 (KRT5) CK+ and CK- areas following Q3 normalisation.

Tumour-stroma boundary shown in Fig. 1B was chosen as the ROIs for each tissue type. Since the sections covered the tumour-stroma boundary, random ROIs were chosen from each section, with cell-dense areas prioritised. The cellular composition can be segmented into areas of illumination (AOI) by dividing ROIs based on the fluorescent signal of individual morphology marker by filtering the UV-light [18] (Fig. 2A).

### 2.5 NanoString GeoMx Digital Spatial Profiler

The epithelial cell marker pan-cytokeratin (PanCK) was used to identify tumour cells in the samples. PanCK-positive or PanCK-negative cells were profiled individually [18] (Fig. 2 B &C).

### 2.6 Histology

Formalin fixed 3D samples were processed overnight using a processor (Thermo Fisher Scientific, Loughborough, UK). The next steps were embedding and sectioning into 5 μm sections. The sections were mounted to Superfrost Plus Slide and the slides were baked at 64 °C for 2 h and then deparaffinised. This was followed by Haematoxylin and eosin (H&E) staining and application of mounting medium for imaging.

### 2.7 Assay quality control and statistical analysis

All steps of the assay control and statistical analysis were conducted on GeoMx Digital Spatial Profiler (DSP) Software and Phyton. The sequencing quality is determined based on sufficient saturation and sensitivity of low expressors. Initially raw probe counts were assessed for sequencing quality control (QC) where all of the under-sequenced samples from AOI count analysis were eliminated from the following QCs. The next step was probe QC which is to target mRNAs by multiple probes and the outlier probes were removed. The data was normalised using third quartile (Q3) normalisation. This normalises individual counts to the 75th percentile of signal from their own AOI. The expression levels were presented as counts that quantify RNA level from the readouts of the barcode. The data was evaluated for signal to Limit of Quantitation (LOQ) ratio to test the reliability of the targets. LOQ was calculated as GeoMean (NegProbes) x GeoSD (NegProbes)^2^. Then, the signal from each probe was divided by the LOQ of each AOI.

Normalised data was statistically assessed by *t*-test (non-paired) with the BH test correction type and tested for by factors. To generate gene volcano plots a custom script for volcano plots by GeoMx Script Hub was used. A p-value of <0.05 was considered statistically significant and the log_2_ fold change log_2_(FC) value > 0.5 was considered as the notable fold change. The graphs were plotted as log_2_(FC) as x-axis and adjusted p-value as y-axis. Heatmaps were created in Python using hierarchical clustering to visualise statistically significant group gene expression profiles. All statistically significant genes were included in the heatmaps for each pathway unless stated otherwise. Individual genes were plotted using GraphPad Software (La Jolla, CA, USA) and their statistical tests were completed using the GeoMx Digital Spatial Profiler (DSP) Software. The pathway networks in Supplemental Material were created using DSP Software, Pathway Analysis feature and the networks were presented based on enrichment score.

### 2.8 Selection of genes for pathway panels

All pathway panels were created using set gene lists available in DSP Reactor Target Groups. Invasion pathway gene panel was created from the cell migration genes and included all MMPs. Matrix remodelling pathway gene panel was composed of ‘ECM proteoglycans’, ‘Matrix Remodelling’, and ‘ECM Interactions’ groups that are defined in Reactor Target Groups. Immune system pathway gene panel included all Immune System Reactor Target Group.

## 3 Results

### 3.1 The ameloblastoma transcriptome is altered in the presence of stromal cells

Tumour-stroma models were engineered using AM-1 cells as a central tumour mass. The central tumour mass was surrounded by specific stromal compartments of dense collagen I with either no cells, gingival fibroblasts (HGF) or osteoblasts (hOB) (Fig. 1A). This resulted in 3 sets of cultures to compare, with the acellular stroma essentially acting as a control. The GeoMx Digital Spatial Profiler (DSP) was used to evaluate the effects on tumour cells of adding specific populations of stromal cells. For the profiler, the area covering the tumour mass-stroma boundary was sectioned (Fig. 1B) in order to focus and capture tumour cells invading into the surrounding stroma. In each section, 3 regions of interest (ROIs) were selected for comprehensive cancer transcriptome analysis (Fig. 2A). The ROIs contained both CK+ and CK- cells, and CK + cells were verified with ∼5–6 timers higher *KRT5* expression compared to CK- cells (Fig. 2B&C).

**Fig. 3:**
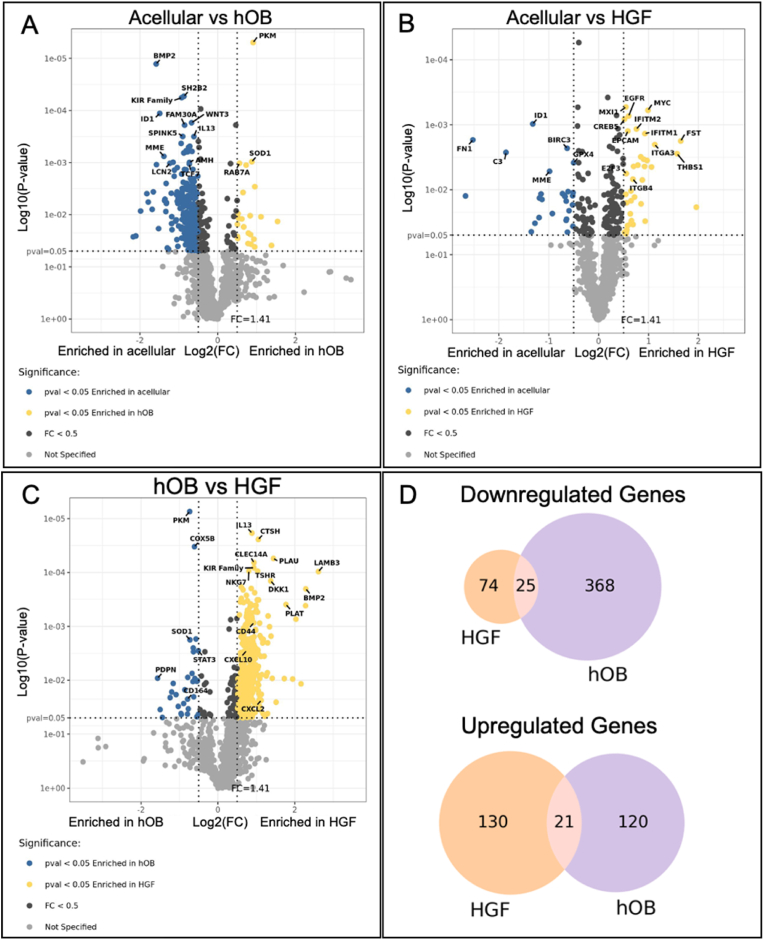
*Volcano plots showing Q3 normalised gene counts that are statistically significant, p-value (pval) < 0.05 and* log_2_(FC) > *0.5 (colour coded as blue or yellow) in 3D Ameloblastoma tumouroids at day 14 in CK* + *cells. Enrichment of genes were compared among (A) acellular stroma versus (vs) human osteoblast (hOB) stroma, (B) acellular stroma vs human gingival fibroblast (HGF) stroma, and (C) hOB stroma versus HGF stroma. Significance was demonstrated in y-axis as log*_*10*_*(P-value) and FC value was presented in x-axis as log*_*2*_*(FC). T-test (non-paired), BH test correction, and tested by Factors. (D) Venn Diagrams of number of genes that are significantly upregulated or downregulated in AM-1 cells with HGF stroma and/or hOB stroma compared with acellular stroma. Gene labels were presented randomly.*

The introduction of different physiologically relevant stromal types induced significant changes in the transcriptome of tumour cells. Out of the 1813 transcriptome genes analysed, 1415 genes were above LOQ *(*Supplemental Figure 2*). Volcano plots were generated to overview the changes in the expression profile of AM-1 cells when interacting with different stroma types. Only genes with log*_*2*_ *fold-change (log*_*2*_*FC) more than 0.5 were further analysed* (Fig. 3 A&B&C)*.* Initial screening of these volcano plots indicated that the introduction of stromal cells in tumour adjacent compartments induced significant changes (45.3 %) in the transcriptome of AM-1 cells compared to acellular stroma. 15.3 % of the whole transcriptome was significantly altered when a fibroblast stroma was introduced to the AM-1 tumour mass and 36.7 % of genes changed with a bone stroma (the hOBs) (Fig. 3). The Venn Diagrams in Fig. 3D demonstrate the number of genes that were significantly downregulated or upregulated with bone stroma and/or fibroblast stroma compared to acellular stroma. The number of genes downregulated with bone stroma was higher than with fibroblast stroma (Fig. 3D).

Considering there were 1415 genes, to better prioritise targets in the case of ameloblastoma the rest of the analysis was segmented based on different signalling pathways. Having a fibroblast stroma or osteoblast stroma caused enrichment in many different pathways and all of these pathways presented in pathway networks (Supplemental Figures 3, 4 and 5). The networks showed greatest enrichment in pathways such as DNA repair, extracellular matrix organisation, DNA replication, immune system, and cell cycle.

### 3.2 Adding stromal cells cause significant changes in ameloblastoma invasion

Invasion is a tumour specific characteristic driven by different pathways, including epithelial to mesenchymal transition (EMT), migration and cell adhesion pathways [19]. Tumour cells, including ameloblastoma cells, are known to invade to their surrounding stroma within the 3D tumouroid [11]. Since the sectioned area covered the tumour-stroma boundary, the AM-1 cells that were migrating into the surrounding stroma were spatially profiled.

**Fig. 4:**
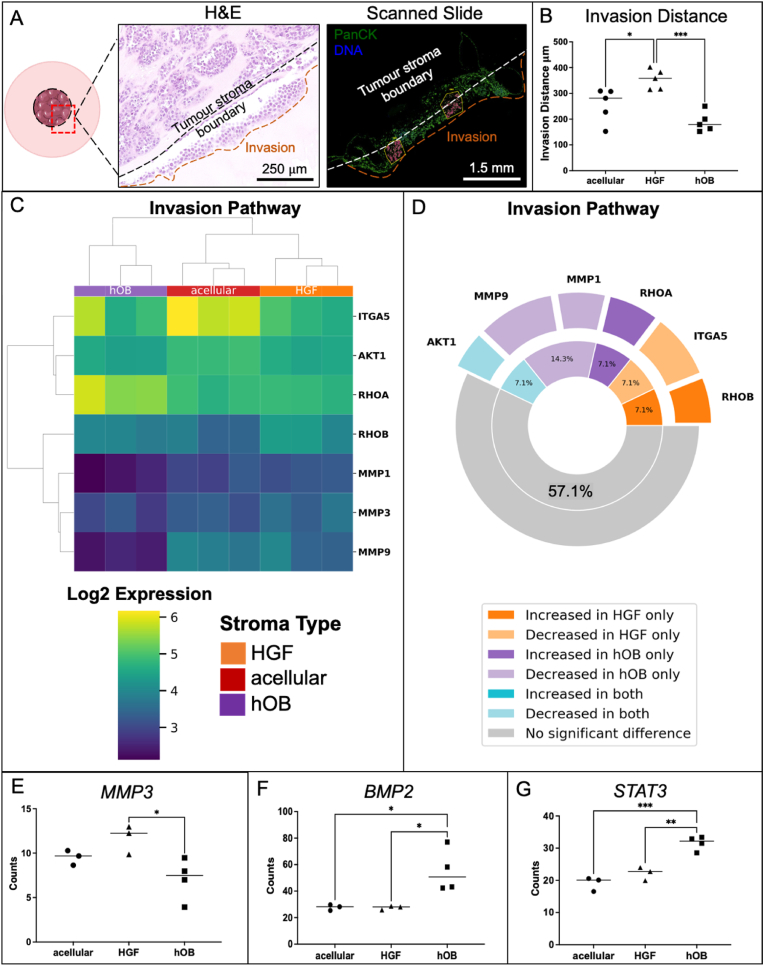
Invasion of AM cells to different stroma. (A) Invasion of AM-1 cells within the 3D tumouorids shown from H&E-stained samples and GeoMx Profiler scanned samples, green = Pan Cytokeratin (PanCK), blue = DNA. scale bars = 250 μm and 1.5mm respectively. White lines = tumour mass and stroma boundary, orange lines = invasion of tumour cells to the surrounding stroma. (B) Invasion distance of AM-1 cells from the tumour mass to acellular, HGF, and hOB stroma at day 14. (C) **Correlation heatmap of differentially expressed genes in invasion pathway** in AM-1 tumouroids with acellular, HGF, and hOB. Heatmap presents log_2_ change from Mean. T-test (non-paired), BH test correction, and tested by Factors. (D) Plot showing significant gene change. The inner ring represents the percentage of pathway genes significantly changed in the presence of each stroma type. The outer ring indicates the relative fold change in the gene expression observed for each gene in the subgroups. Gene counts of (E) MMP3, (F) BMP2, and (G) STAT3 in AM-1 tumouroids with acellular, HGF and hOB stroma. One-Way ANOVA, Dunnet's Post Hoc; p-values 0.05 < *, 0.005 < **, and 0.0005 < ***. Diagrams were created using Smart Servier Medical Art.

The invasion of AM-1 cells to their surrounding stroma by day 14 was shown in Fig. 4A. The invasion data supports previous findings on the invasion of AM-1 cells into different stromal compartments, namely acellular, fibroblast stroma (HGF) and bone stroma (hOBs). AM-1 cell invasion was significantly higher where a fibroblast stroma was present (355 ± 39 μm) compared to an acellular stroma (255 ± 66 μm, p < 0.05) or indeed to a bone stroma (189 ± 39 μm, p < 0.0005) (Fig. 4B). The statistically significant change in gene expression (p < 0.05) of the invasion of ameloblastoma cells into either an acellular stroma or a bone stroma was 28.6 %, between an acellular stroma and fibroblast stroma it was 21.4 %. These changes indicated that adding stromal cells induced a decrease in specific invasion genes *(*Supplemental Table 1*) (*Fig. 4*D).*

*A heatmap covering EMT, cell migration and MMPs was generated for AM-1 (ameloblastoma cells), which were the PanCK* + *tissue segments. Different stroma types directly impacted the expression profiles of AM-1 tumour cells. AM-1 tumouroids with bone stroma exhibited the greatest change in invasion markers compared to other stroma types (*Fig. 4 *C&D).* Introducing stromal cells induced an enrichment of certain migration genes. For example, Rho-associated protein kinase (*ROCK1)* was enriched where a bone stroma was present and this has previously been found to be overexpressed in cell migration and invasion in neoplasms [20]. One of the main invasion markers matrix metalloproteinases 3 (*MMP3*) [21] was ∼2-fold upregulated where a fibroblast stroma was present compared to a bone stroma, which correlates with the invasion distance data (Fig. 4E). The expression of other invasion markers, *MMP1* and *MMP9*, were higher where a fibroblast stroma was present compared to a bone stroma. Interestingly, there was no significant change in some of the other main invasion markers such as *MMP11* and *MMP7* with different stroma types (Fig. 4C).

Besides cell migration and adhesion targets, other invasion markers were also assessed. The invasion marker, bone morphogenic protein *(BMP2)* [22] was ∼2-fold upregulated in AM-1 tumouroids with a bone stroma compared to a fibroblast stroma (p < 0.05) and an acellular stroma (p < 0.05) (Fig. 4F). The invasion and metastasis marker, the signal transducer and activator of transcription 3 *(STAT3)* [23] was significantly upregulated with a bone stroma compared to a fibroblast stroma (p < 0.005) *(*Fig. 4*G).*

### 3.3 Matrix remodelling ability of the tumour cells is dependent upon their stroma

Tumour cells rely on the interactions with their ECM during cell migration and invasion. There are ∼300 unique matrix macromolecules such as collagens, proteoglycans and glycoproteins including laminins. Remodelling of the basement membrane, which is mainly composed of collagen IV and laminins, is essential for tumour invasion [24]. The four main mechanisms of tumorigenic ECM remodelling are ECM deposition, chemical modifications, proteolytic degradation and force-mediated physical remodelling [24]. Therefore, the next set of analyses was based on changes related to matrix remodelling targets.

**Fig. 5:**
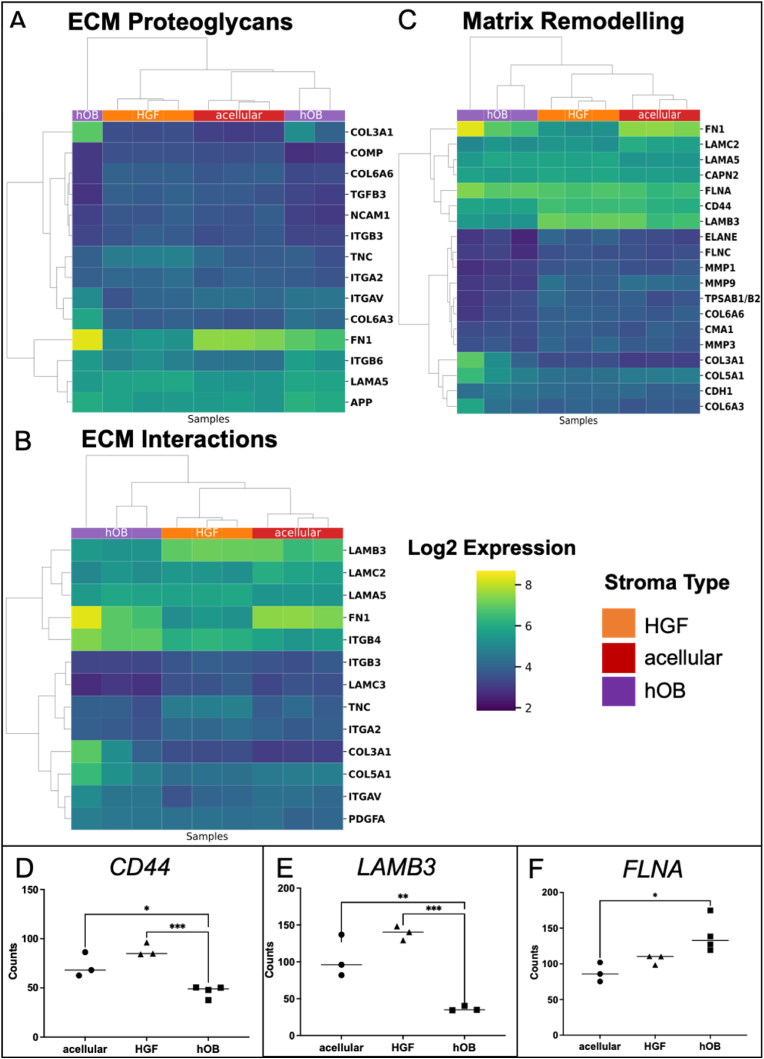
Change in matrix remodelling genes with the introduction of different stromal types. **Correlation heatmap of differentially expressed genes in** (A) ECM proteoglycan pathway, (B) ECM interactions pathway, and (C) matrix remodelling pathway in AM-1 tumouroids with acellular, HGF, and hOB. Heatmap presents log_2_ change from Mean with hierarchical clustering. T-test (non-paired), BH test correction, and tested by Factors. Gene counts of (D) CD44, (E) LAMB3, and (F) COL27A1 in AM-1 tumouroids with acellular, HGF and hOB stroma. One-Way ANOVA, Dunnet's Post Hoc; p-values 0.05 < *.

**Fig. 6:**
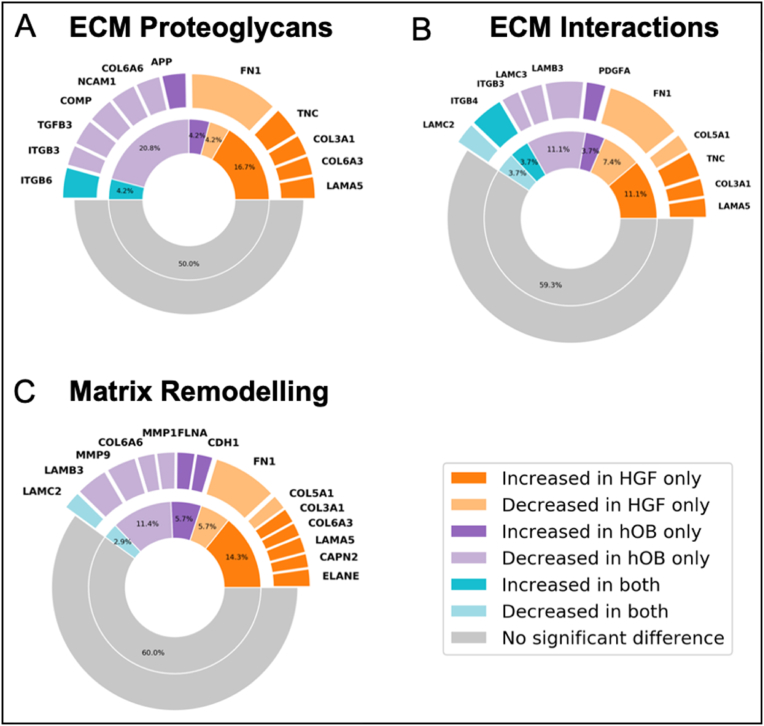
Plots showing significant gene change for (A) ECM Proteoglycans, (B) ECM Interactions, and (C) Matrix Remodelling. The inner ring represents the percentage of pathway genes significantly changed in the presence of each stroma type.

The heatmap for the expression of ECM Proteoglycans targets in AM-1 cells in tumouroids showed varied enrichment levels in particular with the addition of a bone stroma. Certain ECM proteoglycan genes such as the metastatic marker amyloid precursor protein *(APP)* [25] was significantly upregulated in the presence of a bone stroma. AM-1 cells in the presence of a fibroblast stroma exhibited the greatest enrichment for ECM proteoglycan targets compared to a bone stroma and an acellular stroma (Fig. 5A). 83.3 % of the genes altered in AM-1 cells within a fibroblast stroma were upregulated, where this percentage of upregulated genes was only 28.6 % when a bone stroma was present. Overall, 50 % of the ECM proteoglycan genes were altered by introducing stromal cells *(*Supplemental Table 2 *and* Fig. 6*A). A similar pattern was observed in ECM interaction targets as well as the matrix remodelling targets (*Fig. 5 *B&C).* Introduction of a fibroblast stroma induced a 57.1 % increase among the altered ECM interaction pathway genes in AM-1 tumour cells, whereas in the presence of a bone stroma there was only a 33.3 % increase. The percentage of genes altered, specifically the ECM interaction pathways, in AM-1 cells by the addition of stromal cells was 40.7 % *(*Supplemental Table 3*)* and in the matrix remodelling pathway this *was* 40.0 % *(*Supplemental Table 4*).* Addition of either a bone or fibroblast stroma caused significant upregulation of ECM genes involved in tumour progression [26,27] including Integrin αvβ6 *(ITGB6)* and *ITGB4 (*Fig. 6 *A&B).*

The epithelial-to-mesenchymal transition marker that is highly expressed in primary and metastatic cancers, *CD44* [28], was significantly upregulated where a fibroblast stroma was present compared to a bone stroma (∼2-fold) (p < 0.0005) (Fig. 5D). The expression of a metastasis marker, the laminin subunit beta-3 *(LAMB3,)* was ∼3-fold higher with the fibroblast stroma compared to a bone stroma (p < 0.0005) (Fig. 5E). Among matrix remodelling targets, the expression of collagen genes such as collagen type 3 Alpha 1 Chain (*COL3A1)* and *COL6A3* in AM-1 tumouroids with a fibroblast stroma were higher compared to acellular stroma and bone stroma (Fig. 5C). The matrix remodelling gene Filamin A *(FLNA)* [29], was significantly upregulated where a bone stroma was present compared to an acellular stroma (p < 0.05) (Fig. 5F).

### 3.4 Increasing stromal complexity induced enrichment of immune markers by AM-1 tumour cells

**Fig. 7:**
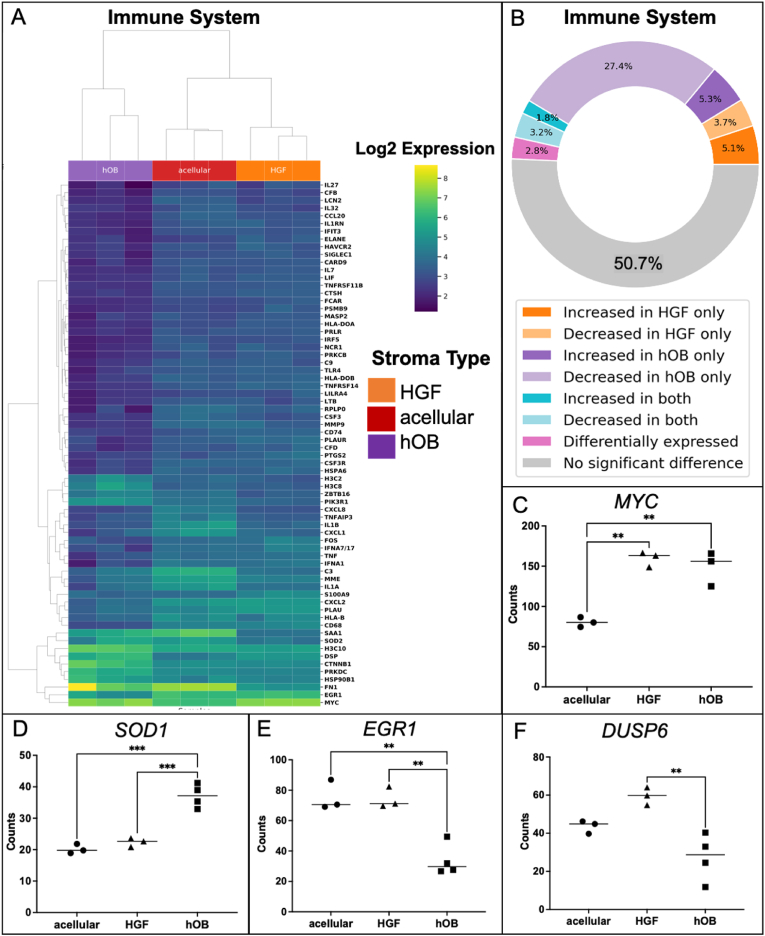
Change in immune system genes with the introduction of different stromal types. **Correlation heatmap of differentially expressed genes** (A) immune system pathway in AM-1 tumouroids with acellular, HGF, and hOB. Data was presented as Log_2_ changes from Mean. T-test (non-paired), BH test correction, and tested by Factors. (B) Plot showing significant gene change. The ring represents the percentage of pathway genes significantly changed in the presence of each stroma type. Gene counts of (C) MYC, (D) SOD1, (E) EGR1 and (F) DUSP in AM-1 tumouroids with acellular, HGF and hOB stroma. One-Way ANOVA, Dunnet's Post Hoc; p-values 0.05 < *, 0.005 < **, and 0.0005 < ***.

The ECM remodelling by tumour cells influences the inflammatory tumour environment. Components of the ECM act as inflammatory stimuli and drive immune response [24]. Therefore, from the cancer transcriptome, the immune pathways have a significant role in understanding how tumour cells communicate with their stroma. The heatmap of the immune system pathway indicate enrichment of the targets by AM-1 cells in the presence of both a bone stroma and a fibroblast stroma compared to an acellular stroma (Fig. 7 A).

Addition of either a fibroblast or bone stromal compartment to the AM-1 tumouroid induced changes in the expression of 49.3 % of genes in the immune system pathway compared to an acellular stroma *(*Supplemental Table 5*).* In particular, the fibroblast stroma induced a significant upregulation in 54.9 % of the significantly changed immune system pathway genes in AM-1 cells compared to where an acellular stroma was present. Of the genes that were differentially expressed in the presence of different stromal compartments, 85.05 % were higher where a fibroblast stroma was present. Whereas the presence of a bone stroma resulted in significant changes to 40.6 % of all immune genes, of which 81 % were downregulated *(*Supplemental Table 5*)* and (Fig. 7B). The presence of stromal cells resulted in the upregulation of certain oncogenes and downregulated the expression of tumour suppressors, which are part of the immune system pathways. The oncogene, *MYC* [30] was ∼2-fold upregulated in AM-1 tumouroids with a fibroblast stroma (p < 0.005) or a bone stroma (p < 0.005) compared to an acellular stroma (Fig. 7C). The expression of another oncogene, Superoxide Dismutase 1 *(SOD1)* [31] was significantly higher in AM-1 cells cultured with a bone stroma compared to an acellular stroma (Fig. 7D). The tumour suppressor markers early growth response 1 (EGR1) (Fig. 7E) and dual specificity phosphatase 6 (DUSP6) (Fig. 7F) [32] were downregulated by ∼2-fold in the presence of a bone stroma compared to a fibroblast stroma (p < 0.005). The expression of EGR1 was also significantly lower where a bone stroma was present compared to an acellular stroma (p < 0.005) (Fig. 7E).

## 4 Discussion

Developing 3D models that recapitulate tumour-stroma interactions are essential for modelling the biomimetic tumour microenvironment *in vitro*. 3D tumouroid models allow compartmentalisation where different cell types can be added to distinct compartments to increase model complexity. In these complex tumouroids, mixed cell populations are pooled and then analysed for gene and protein markers [11,33]. To date, spatial profiling of the multicompartment 3D models have not been explored. It is difficult to study how the introduction of specific stromal compartments alters or affects tumour cells. This study is the first to capture the region of interest within a bioengineered 3D model and specifically analyse the tumour-stroma boundary.

Bioengineered tumouroids are the first 3D *in vitro* tumour model to be analysed using spatial transcriptomics. Key to this is the sample preparation of spatial transcriptomics which requires embedding and sectioning of the sample. There are limited 3D models that can be processed for histology [11,34,35]. Frozen sectioning is used for spheroid models [36]. It is also important to consider the fact that the cell number of 3D models are much lower than normal tissue, and this makes it difficult to section the area of interest. This study described a novel protocol for how to utilise 3D tumouroids for spatial transcriptomics analysis. Within tumouroids the tumour borders of the tumour mass are visible by eye, thus locating the tumour stroma boundary within the tumouroids is possible during sectioning.

This study assessed changes in the cancer transcriptome of the tumour cells within the 3D tumouroids as the stromal complexity increased. The regions of interests were the boundary between the ameloblastoma tumour mass and the surrounding stromal compartment. By focusing on this region, it was possible to understand the changes in the tumour cells that are invading into the surrounding stroma. The volcano plots generated from the spatial data showed that there are changes in the expression of specific genes in AM-1 tumour cells with different stroma (Fig. 3).

Following generation of volcano plots and heat maps, several pathways from the cancer transcriptome were identified as being significantly altered by the introduction of different stroma. Initially, the changes in the invasion pathway targets were compared to the invasion distance data. The introduction of a fibroblast stroma caused upregulation in invasion markers including MMP3 [21] and this upregulation was in line with the observed and quantified increase in the invasion distance of AM-1 tumouroids where a fibroblast stroma was present compared to an acellular stroma and a bone stroma. MMPs are well-studied in the case of ameloblastoma, however there is not much data on MMP-3 and most of the reported literature is on MMP-2, -7, and -9 [37,38]. Therefore, this study suggests MMP-3 as an invasive marker for ameloblastoma. The reason for not observing a change in other known MMPs may be because some of these are involved in the earlier stages of invasion. Although AM-1 cells invaded the shortest distance into a bone stroma, several invasion genes and metastasis genes such as RHOA, BMP2 and STAT3 were upregulated. These markers are associated with later migration compared to MMPs. For example, BMP-2 has been associated with tumour progression in the late stages of gastric cancer [39].

Invasion and matrix remodelling are closely linked [24], therefore it is essential to understand how genes in specific pathways change by adding different stromal cells. Tumour cell-influenced matrix remodelling prepares the TME for tumorigenesis and metastasis. Tumour cells can cause direct or indirect breakdown of the ECM to invade or migrate quicker [24]. ECM features such as porosity, crosslinking and density also affect cell migration [40].

All these properties were altered with the addition of either a bone or fibroblast stromal compartment within AM-1 tumouroids. Fibroblast stroma led to the greatest enrichment in ECM remodelling targets compared to bone stroma (Supplemental Table 4). This clearly correlated with measured invasion into the fibroblast stroma (Fig. 4B). Collagen alignment aids the invasion of tumour cells and collagen chains modulate tumour formation and metastasis [41,42]. EMT enhances tumour cell mobility, invasion, and metastasis, therefore upregulation of *CD44* with a fibroblast stroma (Fig. 5D) is expected when taking into consideration the corresponding invasion distance data (Fig. 4B). Our data shows that the bone stroma induce changes in AM-1 cells in some of the matrix remodelling genes such as *COL27A1,* a gene that is overexpressed by tumour cells and induces ECM production [43]. This might be associated with slow migration of the AM-1 cells into the bone stroma compared to into a fibroblast and acellular stroma.

Finally, introducing stromal cells induced high enrichment in pathways related to immune system (Fig. 6A). Similar enrichment has recently been reported in invasive seminoma germ cell tumours [44]. Upregulation of the oncogene *MYC* in the presence of either stromal cell, indicated the importance of increasing the complexity of 3D models. Interestingly, the bone stroma induced the downregulation of tumour suppressor genes within AM-1 cells. It is known that tumour enrichment of tumour markers are linked to enrichment of immune markers [7].

This study highlights how tumour cells are affected and influenced by specific stromal cells within their micro-environment. As tumour cells come into contact with a stromal cell or sense changes in the stroma, significant changes occur.

The fact that it is possible to process 3D tumouroids using methods similar to tissue samples with optimised protocols along with the ability to conduct spatial analysis, highlight the power of this bioengineered 3D model. The spatial transcriptome data has been used to profile the tumour-stroma boundary within the 3D models. Prior to this work, it was not possible to dissect out and analyse CK + cells or from a mixed cell population in 3D tumouroid. This technique allows for the capture of a region of interest, namely the tumour-stroma boundary. This work will guide future studies that are interested in the applications of spatial transcriptomics in *vitro* 3D models.

## 5 Limitations

The main limitation is that the GeoMx DSP does not have the same specificity as the single cell sequencing since the platform has 1–10 cells in each spatial spot for analysis. Future steps, will include comparing the transcriptome of ameloblastoma in different locations within the tumouroids, for instance deep within the hypoxic core of the tumour mass, or invasive cells within the stroma. The spatial analysis of the whole 3D tumouroid model will enable us to understand the differences in expression patterns within the tumouroid.

## Ethics approval and consent to participate

Not Applicable.

## Consent to publish

Not Applicable.

## Availability of data and materials

The authors confirm that data and material in this study are presented in main manuscript.

## Funding

D.B. receives funding from BISS Charitable Foundation. G.A. receives funding from 10.13039/501100000266EPSRC, 10.13039/100015980EP/W522636/1.

## CRediT authorship contribution statement

**Deniz Bakkalci:** Conceptualization, Formal analysis, Investigation, Methodology, Visualization, Writing - original draft, Writing - review & editing. **Georgina Al-Badri:** Data curation, Formal analysis, Methodology, Writing - review & editing, Software. **Wei Yang:** Methodology, Software. **Andy Nam:** Formal analysis, Methodology. **Yan Liang:** Methodology, Software. **Syed Ali Khurram:** Conceptualization. **Susan Heavey:** Conceptualization, Methodology. **Stefano Fedele:** Conceptualization, Resources, Funding acquisition. **Umber Cheema:** Conceptualization, Data curation, Formal analysis, Funding acquisition, Investigation, Project administration, Supervision, Validation, Visualization, Writing - review & editing.

## Declaration of competing interest

The authors declare that they have no known competing financial interests or personal relationships that could have appeared to influence the work reported in this paper.

## Data Availability

No data was used for the research described in the article.

## Abbreviations

AM: Ameloblastoma
AOI: Area of Illumination (AOI)
APP: Amyloid Precursor Protein
BPE: Bovine pituitary extract
BMP2: Bone Morphogenic Protein 2
CK: Cytokeratin
*COL3A1*: collagen type 3 Alpha 1 Chain
DMEM: Dulbecco's modified Eagle medium
DSP: Digital Spatial Profiling
*DUSP6*: Dual specificity phosphatase 6 *(DUSP6)*
ECM: Extracellular matrix
*EGR1*: Early Growth Response
FBS: Foetal bovine serum
HGF: Primary gingival fibroblasts
hOB: Human osteoblasts
IF: Immunofluorescence
*ITGB6*: Integrin αvβ6 *(ITGB6)*
H&E: Haematoxylin and eosin
KRT5: Keratin 5
*LAMB3*: laminin subunit beta-3
LOQ: Limit of Quantitation
MEM: Minimal Essential Medium
MIQE: Minimum Information for Publication of Quantitative Real-Time PCR Experiments
MMPs: Matrix metalloproteinases
M: Molar
Min: Minutes
N.A: Neutralising agent
MMP3: matrix metalloproteinases 3
MRC5: Human lung fibroblasts
PanCK: Pan Cytokeratin
*PTHLH*: Parathyroid Hormone Like Hormone
RANK: The tumour necrosis factor (TNF) superfamily members receptor activator of nuclear factor kappa-B receptor
RANKL: The tumour necrosis factor (TNF) superfamily members receptor activator of nuclear factor kappa-B ligand *(TNFSF11)*
ROCK1: Rho-associated protein kinase
ROI: Region of Interest
RT: Room temperature
SD: Standard deviation
SEM: Standard error mean
SOD1|: Superoxide Dismutase 1
STAT3: the signal transducer and activator of transcription 3
TGF-β: Transforming growth factor
THBS1: Thrombospondin 1
TME: Tumour microenvironment
TNF: Tumour necrosis factor
