# Supplementary material for: Spatial transcriptomic interrogation of the tumour-stroma boundary in a 3D engineered model of ameloblastoma
Source: Mater Today Bio. 2023 Dec 21;24:100923. doi: 10.1016/j.mtbio.2023.100923 (PMC10788620; doi:10.1016/j.mtbio.2023.100923)
Supplement: Multimedia component 1 [file mmc1.docx]

*
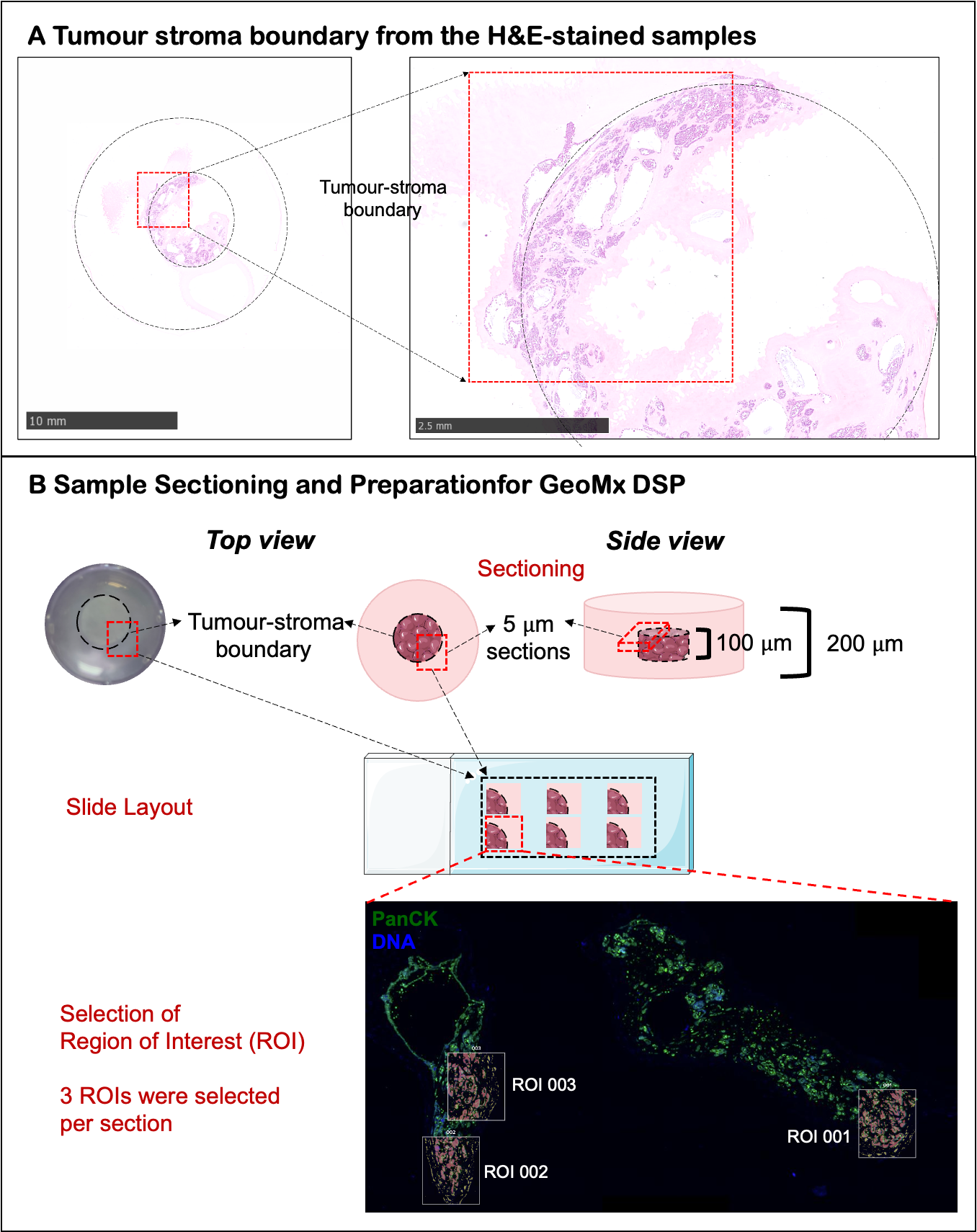
*

*Supplemental Figure 1: Invasion of AM-1 cells within the 3D tumouorids shown to the acellular stroma, shown in H&E-stained samples (A). Sample sectioning and selection of regions of interest for GeoMx Digital Spatial Profiler (DSP) (B). Tumour stroma boundary that were visibly seen by eye was sectioned and the slide layout was shown above. Three regions of interests (ROIs) from each slide were selected for the GeoMx Profiler. Green = Pan Cytokeratin (PanCK), blue = DNA. scale bars = 300 µm. The diagram was created from our previous publication using Smart Servier Medical Art.*

*
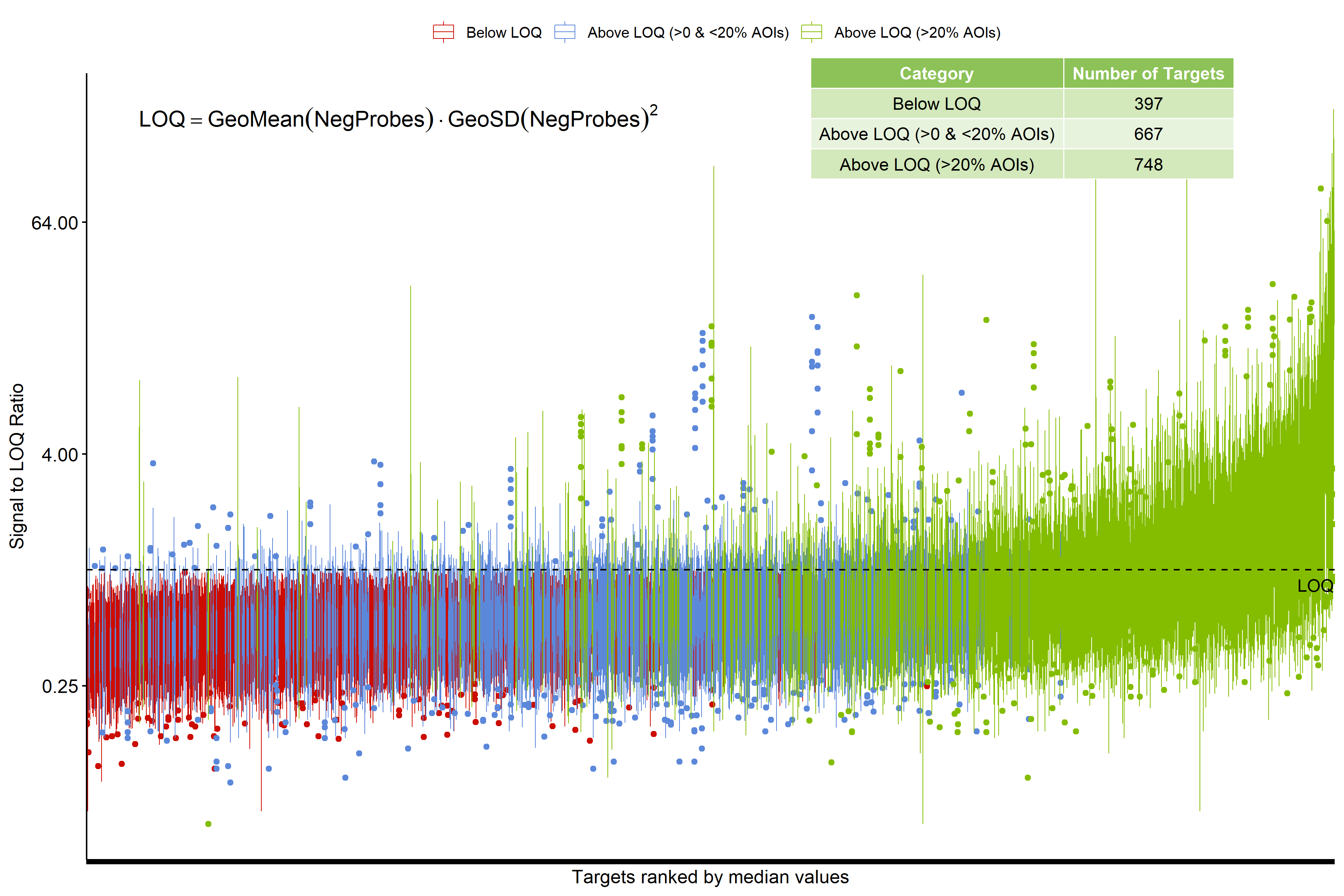
*

*Supplemental Figure 2: Signal to LOQ Ratio performance. Number of targets/genes that were below LOQ, and above LOQ was demonstrated in the table. LOQ was measured from the equation “GeoMean(NegProbes)xGeoSD(NegProbes)^2^ .*


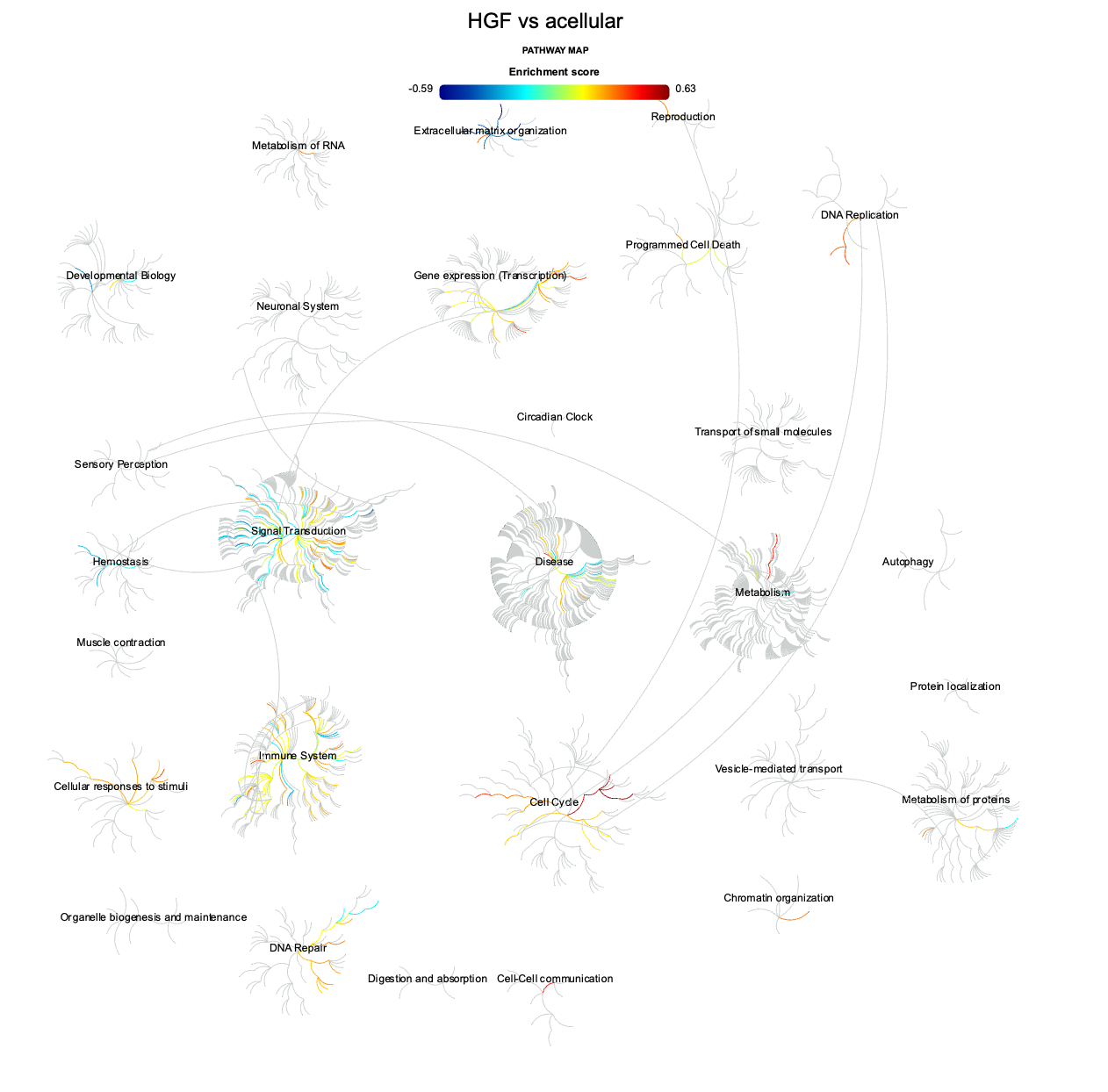


*Supplemental Figure 3: Pathway network representing all pathways affected by fibroblast stroma (HGF) compared to acellular stroma.*


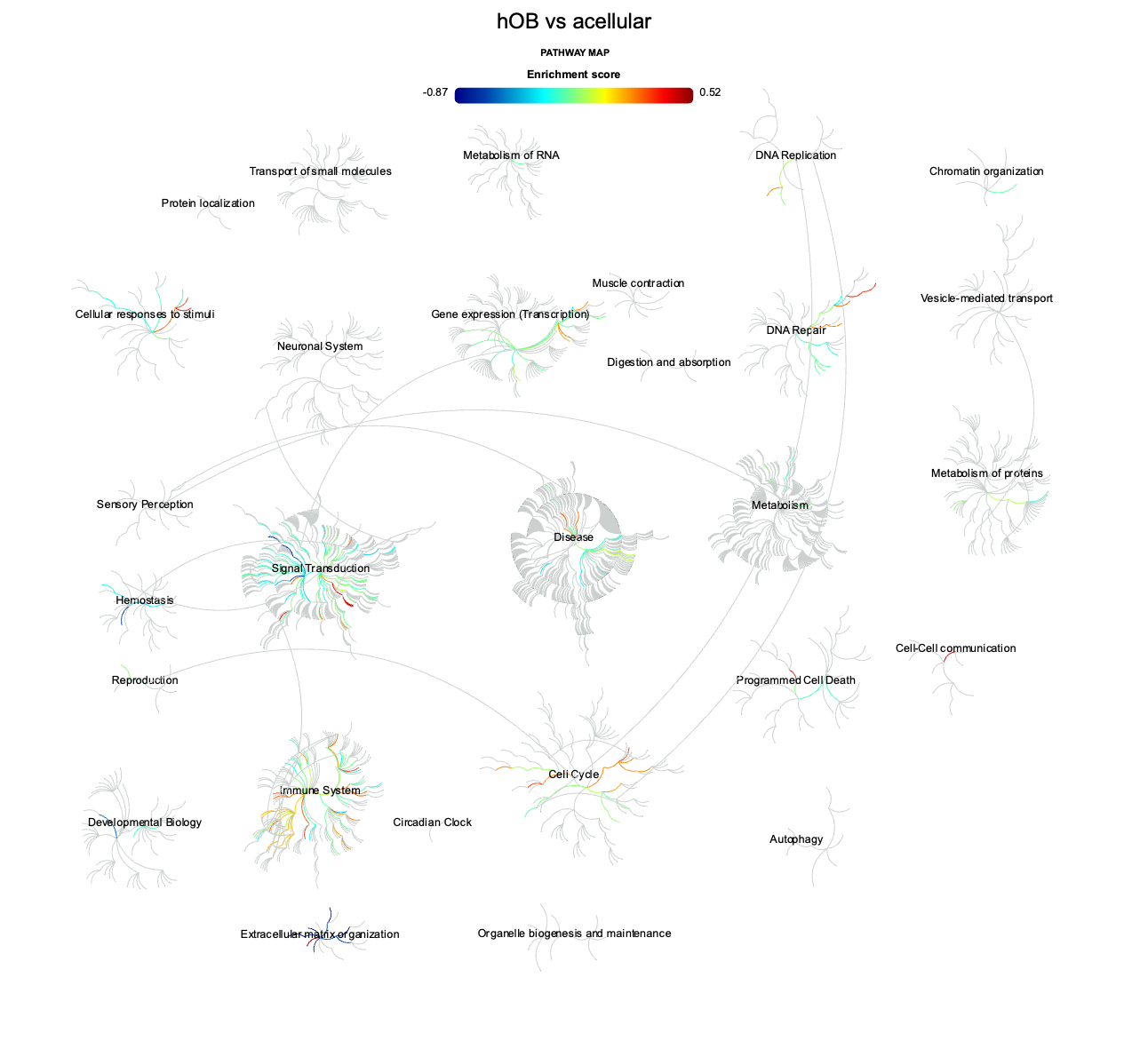


*Supplemental Figure 4: Pathway network representing all pathways affected by osteoblast stroma (hOB) compared to acellular stroma.*


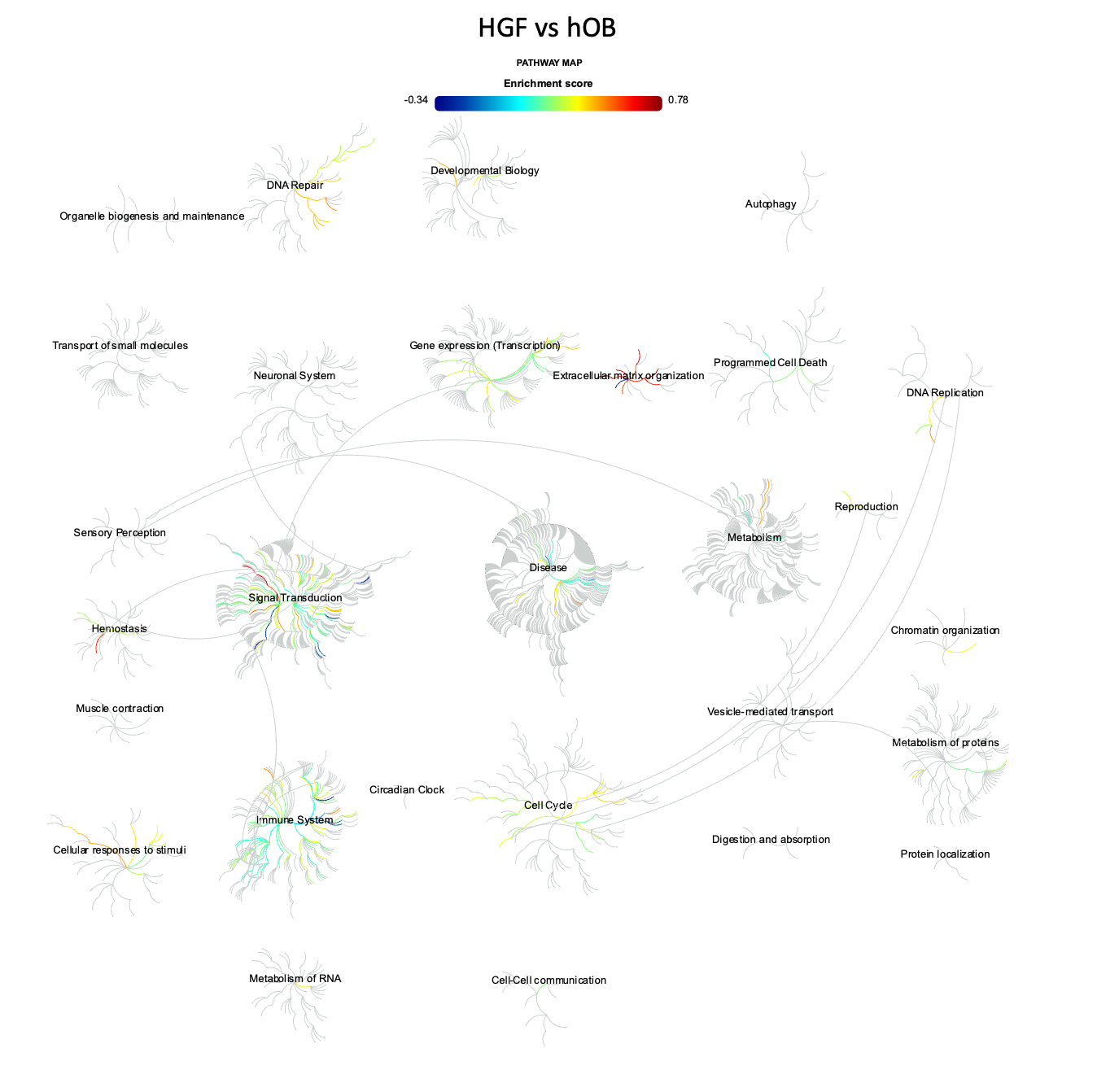


*Supplemental Figure 5: Pathway network representing all pathways affected by fibroblast stroma (HGF) compared to osteoblast stroma (hOB).*

| **Invasion Pathway**  **Total no. genes: 14** | | | |
| --- | --- | --- | --- |
|  | **% of genes changed** | **% of which increased** | **% of which decreased** |
| HGF vs ac | 21.4 | 33.3 (7.1 of total) | 66.6 (14.3 of total) |
| hOB vs ac | 28.6 | 25 (7.1 of total) | 75 (21.4 of total) |
| HGF OR hOB vs ac | 42.9 |  |  |
|  | **% of genes differentially expressed** | **% of which higher with HGF stroma** | **% of which lower with HGF stroma** |
| HGF vs hOB | 28.6 | 75 (21.4 of total) | 25 (7.1 of total) |
|  | **Genes with increased expression (vs ac)** | **Genes with decreased expression (vs ac)** |  |
| HGF | RHOB | ITGA5, AKT1 |  |
| hOB | RHOA | AKT1, MMP11, MMP9 |  |
|  | **Genes with increased expression (vs hOB)** | **Genes with decreased expression (vs hOB)** |  |
| HGF | MMP1, MMP3, MMP9 | RHOA |  |
| Genes with two significances:   - **AKT1** significantly lower in AM-1 cells cultured with either HGF or hOB stroma compared to acellular stroma. - **MMP9** significantly higher in AM-1 cells cultured with HGF compared to both hOB stroma and acellular stroma. - **RHOA** significantly higher in AM-1 cells cultured with hOB compared to both HGF stroma and acellular stroma. | | | |

*Supplemental Table 1: Invasion pathway and genes that are significantly changed between different stroma types. All percentages represent the statistically significant proportion of genes that are changed.*

| **ECM Interactions Pathway**  **Total no. genes: 27** | | | |
| --- | --- | --- | --- |
|  | **% of genes changed** | **% of which increased** | **% of which decreased** |
| HGF vs ac | 25.9 | 57.1 (14.8 of total) | 42.9 (11.1 of total) |
| hOB vs ac | 22.2 | 33.3 (7.4 of total) | 66.7 (14.8 of total) |
| HGF OR hOB vs ac | 40.7 |  |  |
|  | **% of genes differentially expressed** | **% of which higher with HGF stroma** | **% of which lower with HGF stroma** |
| HGF vs hOB | 33.3 | 55.6 (18.5 of total) | 44.4 (14.8 of total) |
|  | **Genes with increased expression (vs ac)** | **Genes with decreased expression (vs ac)** |  |
| HGF | LAMA5, ITGB4, COL3A1, TNC | COL5A1, LAMC2, FN1 |  |
| hOB | ITGB4, PDGFA | LAMC2, LAMC3, LAMB3, ITGB3 |  |
|  | **Genes with increased expression (vs hOB)** | **Genes with decreased expression (vs hOB)** |  |
| HGF | LAMB3, ITGA2, LAMC3, ITGB3, TNC | ITGB4, ITGAV, PDGFA, FN1 |  |
| Genes with two significant differences:   - **ITGB4** was significantly increased in AM-1 cells cultured with HGF stroma and hOB stroma (vs acellular stroma), and was significantly higher with hOB stroma compared to HGF stroma. - **LAMC2** was significantly decreased with either HGF or hOB vs acellular stroma. - **ITGB3, LAMC3, and LAMB3** were significantly decreased with hOB compared to HGF and acellular stroma. - **TNC** was significantly increased with HGF stroma compared to hOB and acellular stroma. - **PDGFA** was significantly increased with hOB stroma compared to HGF and acellular stroma. - **FN1** was significantly decreased with HGF stroma compared to acellular and hOB stroma. | | | |

*Supplemental Table 2: ECM interaction Pathway. Table describing gene alteration among different stroma types. All percentages represent the statistically significant proportion of genes that are changed.*

| **Matrix Remodelling Pathway**  **Total no. genes: 35** | | | |
| --- | --- | --- | --- |
|  | **% of genes changed** | **% of which increased** | **% of which decreased** |
| HGF vs ac | 22.9 | 33.3 (7.1 of total) | 66.6 (14.3 of total) |
| hOB vs ac | 20 | 25 (7.1 of total) | 75 (21.4 of total) |
| HGF OR hOB vs ac | 40.0 |  |  |
|  | **% of genes differentially expressed** | **% of which higher with HGF stroma** | **% of which lower with HGF stroma** |
| HGF vs hOB | 37.1 | 84.6 (31.4 of total) | 15.4 (5.7 of total) |
|  | **Genes with increased expression (vs ac)** | **Genes with decreased expression (vs ac)** |  |
| HGF | ELANE, CAPN2, LAMA5, COL6A3, COL3A1 | COL5A1, LAMC2, FN1 |  |
| hOB | CDH1, FLNA | MMP1, COL6A6, MMP9, LAMB3, LAMC2 |  |
|  | **Genes with increased expression (vs hOB)** | **Genes with decreased expression (vs hOB)** |  |
| HGF | ELANE, MMP1, CAPN2, COL6A6, TPSAB1/B2, MMP9, CMA1, MMP3, LAMB3, CD44, FLNC | CDH1, FN1 |  |
| Genes with two significances:   - **LAMC2** significantly lower in AM-1 cells cultured with HGF stroma and hOB stroma compared to acellular stroma. - **ELANE** and **CAPN2** significantly higher with HGF compared to both hOB and ac - **MMP1, COL6A6, MMP9, LAMB3** all significantly lower with hOB compared to HGF and ac - **CDH1** significantly higher with hOB compared to HGF and ac - **FN1** significantly lower with HGF compared to hOB and ac | | | |

*Supplemental Table 3: Matrix Remodelling Pathway. Genes that are significantly changed between different stroma types. All percentages represent the statistically significant proportion of genes that are changed.*

| **ECM Proteoglycans**  **Total no. genes: 24** | | | |
| --- | --- | --- | --- |
|  | **% of genes changed** | **% of which increased** | **% of which decreased** |
| HGF vs ac | 25.0 | 83.3 (20.8 of total) | 16.7 (4.7 of total) |
| hOB vs ac | 29.2 | 28.6 (8.3 of total) | 71.4 (20.8 of total) |
| HGF OR hOB vs ac | 50.0 |  |  |
|  | **% of genes differentially expressed** | **% of which higher with HGF stroma** | **% of which lower with HGF stroma** |
| HGF vs hOB | 45.8 | 63.6 (29.2 of total) | 36.4 (16.7 of total) |
|  | **Genes with increased expression (vs ac)** | **Genes with decreased expression (vs ac)** |  |
| HGF | LAMA5, ITGB6, COL6A3, COL3A1, TNC | FN1 |  |
| hOB | APP, ITGB6 | COL6A6, NCAM1, COMP, TGFB3, ITGB3 |  |
|  | **Genes with increased expression (vs hOB)** | **Genes with decreased expression (vs hOB)** |  |
| HGF | COL6A6, NCAM1, COMP, TGFB3, ITGA2, ITGB3, TNC | APP, ITGB6, ITGAV, FN1 |  |
| Genes with two significances:   - **ITGB6** significantly higher in AM-1 cells cultured with HGF stroma and hOB stroma compared to acellular stroma, also significantly higher with hOB stroma compared to HGF (ac < HGF < hOB) - **COL6A6, NCAM1, COMP, TGFB3, ITGB3** were all significantly lower with hOB stroma compared to both HGF stroma and acellular stroma. - **TNC** significantly higher with HGF stroma compared to both hOB stroma and acellular stroma. - **APP** significantly higher with hOB stroma compared to HGF stroma and acellular stroma. - **FN1** significantly lower with HGF stroma compared to both hOB stroma and acellular stroma. | | | |

*Supplemental Table 4: ECM Proteoglycans Pathway. Genes that are significantly changed between different stroma types. All percentages represent the statistically significant proportion of genes that are changed.*

| **Immune Pathway**  **Total no. genes: 493** | | | |
| --- | --- | --- | --- |
|  | **% of genes changed** | **% of which increased** | **% of which decreased** |
| HGF vs ac | 16.6 | 54.9 (9.1 of total) | 45.1 (7.5 of total) |
| hOB vs ac | 40.6 | 19.0 (7.7 of total) | 81 (32.9 of total) |
| HGF or hOB vs ac | 49.3 |  |  |
|  | **% of genes differentially expressed** | **% of which higher with HGF** | **% of which lower with HGF** |
| HGF vs hOB | 55.6 | 85.0 (47.3 of total) | 15.0 (8.3 of total) |
| Number of significant genes: 331 | | | |

*Supplemental Table 5: Immune Pathway. Table representing % of genes that are changed among different stroma. All percentages represent the statistically significant proportion of genes that are changed.*
